# Real-time monitoring of the amyloid β_1–42_ monomer-to-oligomer channel transition using a lipid bilayer system

**DOI:** 10.1093/pnasnexus/pgad437

**Published:** 2023-12-14

**Authors:** Yuri Numaguchi, Kaori Tsukakoshi, Nanami Takeuchi, Yuki Suzuki, Kazunori Ikebukuro, Ryuji Kawano

**Affiliations:** Department of Biotechnology and Life Science, Tokyo University of Agriculture and Technology, Tokyo 184-0011, Japan; Department of Biotechnology and Life Science, Tokyo University of Agriculture and Technology, Tokyo 184-0011, Japan; Department of Biotechnology and Life Science, Tokyo University of Agriculture and Technology, Tokyo 184-0011, Japan; Department of Chemistry for Materials, Graduate School of Engineering, Mie University, Mie 514-0102, Japan; Department of Biotechnology and Life Science, Tokyo University of Agriculture and Technology, Tokyo 184-0011, Japan; Department of Biotechnology and Life Science, Tokyo University of Agriculture and Technology, Tokyo 184-0011, Japan

## Abstract

This study describes the observation of the transformation of monomeric amyloid β_1–42_ (Aβ42) into oligomers in a lipid membrane utilizing a lipid bilayer system for electrophysiological measurement. The relevance of oligomers and protofibrils in Alzheimer's disease (AD) is underscored given their significant neurotoxicity. By closely monitoring the shift of Aβ42 from its monomeric state to forming oligomeric channels in phospholipid membranes, we noted that this transformation transpired within a 2-h frame. We manipulated the lipid membrane's constitution with components such as glycerophospholipid, porcine brain total lipid extract, sphingomyelin (SM), and cholesterol (Chol.) to effectively imitate nerve cell membranes. Interesting findings showcased Chol.'s ability to foster stable oligomeric channel formation in the lipid membrane, with SM and GM1 lipids potentially enhancing channel formation as well. Additionally, the study identified the potential of a catechin derivative, epigallocatechin gallate (EGCG), in obstructing oligomerization. With EGCG present in the outer solution of the Aβ42-infused membrane, a noteworthy reduction in channel current was observed, suggesting the successful inhibition of oligomerization. This conclusion held true in both, prior and subsequent, stages of oligomerization. Our findings shed light on the toxicity of oligomers, promising invaluable information for future advancements in AD treatment strategies.

**Significance Statement**

Given the crucial role of amyloid β_1–42_ (Aβ42) oligomerization in the progression of Alzheimer's disease (AD), understanding this process at the molecular level is paramount. This study provides insights into the modulation of this oligomerization by different lipid components in the membrane, potentially paving the way for therapeutic strategies. Further, the identification of a compound capable of impeding Aβ42 oligomerization could herald a significant meaning in the treatment of AD. These findings underscore the importance of continued exploration in this field, as it could lead to useful advances in AD therapeutics.

## Introduction

Alzheimer's disease (AD) is a neurodegenerative disease caused by synaptic loss and neuronal death (1, 2). Amyloid beta (Aβ) is a fragment of amyloid precursor protein (APP), a type I single-pass transmembrane protein with a large extracellular domain, which is implicated in the pathogenesis of AD. Two secretases, β-secretase and ɤ-secretase, cleave an APP to form Aβ fragments with varying lengths (3–5). Amyloid β_1–42_ (Aβ42) is considered to be highly cytotoxic (6), with the monomers aggregating to form insoluble fibrils through oligomeric intermediate states (7). Although Aβ42 fibrils are a major component of senile plaques, their toxicity to neurons would not be high; the oligomeric structure including the protofibril is considered to have a high toxicity (8). Intermediate Aβ42 oligomers/protofibrils have been detected in the brain tissue of AD patients, and nanomolar concentrations of this oligomer solution cause cell death in mature neurons (9). Different types of Aβ assembles, ranging from dimers to large molecular-weight oligomers consisting of >50 monomers, have been described in detail in several studies (10–12). To target this species, lecanemab, a monoclonal antibody that recognizes the protofibrils and prevents the Aβ42 deposition, has recently been approved and used for the treatment of AD (13). Certain studies have also shown the neurotoxicity of specific oligomeric species, and their determination is important for understanding and treating of AD (14).

Various hypotheses have been reported as to the mechanism of neuronal toxicity of the Aβ42 oligomer, including channel formation (15), mitochondrial damage (16), and an increase in hyperphosphorylated tau protein (17). Among them, the channel formation of oligomers in cell membranes is a simple and possible mechanism; the Aβ42 oligomers form channels in the neuronal membrane to increase ion influx, leading to neuronal death by disrupting the intracellular ionic equilibrium (18–21). Several methods for observing the channel formation have been proposed, such as fluorescence measurement of the calcium flux through the channel (22, 23), direct observation of the channel using atomic force microscopy (AFM) (24), and electrophysiological measurements of ion flux through the channels (15, 25). In addition, NMR (26) and molecular dynamic (MD) simulations (27) have also been reported, which estimate the channel structure of Aβ42 in membranes. Although various techniques have demonstrated that Aβ42 oligomers form channels in membranes and increase ion influx, the mechanism of Aβ42 monomer aggregation into channels with a lipid membrane remains incompletely understood. This is due to the limitations of conventional studies, which have relied on preprepared Aβ42 oligomers and have not explored the monomer-to-oligomer transformation with lipid membranes in detail, primarily due to the difficulties associated with kinetic experiments.

**Fig. 1.**
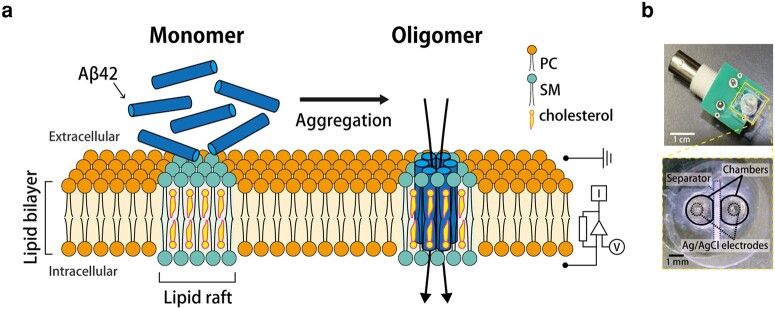
a) An image of channel formation of an Aβ42 oligomer in a planar lipid bilayer membrane. The Aβ42 monomers aggregate into oligomers, forming channels in the membrane and increasing ion influx into the nerve cell. Ions that pass through the Aβ42 channels in the membrane were detected by channel current measurements. b) A photograph of the microdevice for the droplet contact method. The microdevice has two chambers and Ag/AgCl electrodes separated by a separator.

In this study, we report the oligomerization of the Aβ42 monomers, observed from channel current signals using a microfabricated device, which can prepare a stable lipid bilayer (Fig. 1). The obtained channel current signals are identified as the oligomer- or monomer-induced signals, with the signal classification in accordance with our previous reports using antimicrobial peptides (AMPs) (28–30). We also estimate the channel formation in several different types of phospholipid membranes, to imitate the nerve cell membrane using glycerophospholipid (PC), brain total lipid extract from porcine (BTLE), sphingomyelin (SM), and cholesterol (Chol.). The aggregation state of Aβ42 was confirmed by additional methods including western blotting (WB) analysis, thioflavin T (ThT) fluorescence measurement, and AFM observation. Furthermore, based on the signal estimations of monomer-to-oligomer transitions, we aimed to investigate the inhibitory effects of Aβ42 oligomerization using our system. To achieve this, we introduced epigallocatechin gallate (EGCG), a well-known inhibitor of Aβ42 aggregation (31), into the outer solution of the lipid membrane containing Aβ42 monomers and subsequently observed the effects of EGCG on the channel-forming activity of Aβ42 within the membrane. Our system not only enables us to observe the formation of Aβ42 oligomer channels but also facilitates the screening of potential inhibitors of Aβ42 oligomerization, even when embedded within the lipid membrane.

## Experimental

### Reagents and chemicals

In this study, we used the following reagents: 1,2-diphytanoyl-*sn*-glycero-3-phosphocholine (Avanti Polar Lipids, Inc., Alabaster, AL, USA); 1,2-dioleoyl-*sn*-glycero-3-phosphocholine (DOPC; Avanti Polar Lipids); SM (brain, porcine; Avanti Polar Lipids); BTLE (Avanti Polar Lipids); *n*-decane (Wako Pure Chemical Industries, Ltd., Osaka, Japan); Chol. (Sigma-Aldrich Co., St Louis, MO, USA); dimethyl sulfoxide (DMSO; Wako Pure Chemical Industries); 1,1,1,3,3,3-hexafluoro-2-propanol (HFIP; Wako Pure Chemical Industries); sodium chloride (NaCl; Kanto Chemical Co., Inc., Tokyo, Japan); potassium chloride (KCl; Kanto Chemical); sodium hydrogen phosphate (Na_2_HPO_4_; Kanto Chemical); and potassium dihydrogen phosphate (KH_2_PO_4_; Kanto Chemical). Buffered electrolyte solutions were prepared from ultrapure water, which was obtained from a Milli-Q system (Millipore, Billerica, MA, USA). Aβ42 was purchased from Peptide Institute, Inc. (Osaka, Japan). Sample buffer solution with reducing reagent (6×) for SDS–PAGE (Nacalai Tesque, Kyoto, Japan) and Tween20 (Kanto Chemical) and skim milk (Wako Pure Chemical Industries) were used as a solution for WB. Anti–N terminus of Aβ end-specific, mouse monoclonal antibody, 82E1 was purchased from Immuno-Biological Laboratories Co, Ltd. (Gunma, Japan), and horseradish peroxidase (HRP)-conjugated antimouse IgG was obtained from Promega Corporation (Tokyo, Japan). ThT was purchased from Sigma-Aldrich. (-)-EGCG was purchased from Tokyo Chemical Industry Co., Ltd. (Tokyo, Japan).

### Preparation of Aβ42 monomers and oligomers

Regarding the previous study (32), first, 1 mg of powdered Aβ42 was dissolved in HFIP to a final concentration of 1 mM and gently agitated on ice for 10 min. The clear solution containing Aβ42 was quickly aliquoted in Protein LoBind Tubes (Eppendorf, Germany). Next, HFIP was removed from the solution containing Aβ42 under vacuum using a CC-105 centrifugal concentrator and TU-500 cooling trap (TOMY SEIKO Co., Ltd., Tokyo, Japan), and the resulting Aβ42 was stored at −20°C. The Aβ42 aliquots were carefully and completely resuspended to 1 mM in DMSO and sonicated for 3 min (6 cycles of 30 s sonication with 30 s stop) using BIORUPTOR (Cosmo Bio Co., Ltd., Tokyo, Japan). Samples were stored at −80°C if necessary. Immediately prior to use, samples were diluted in PBS buffer solution (137 mM NaCl, 2.7 mM KCl, 8.1 mM Na_2_HPO_4_, 1.4 mM KH_2_PO_4_, and pH 7.4) to 10 µM and pipetted. The oligomer was prepared by incubating the monomer solution in a tube for 4 h at 37°C.

### Preparation of the microdevice for electrophysiological measurements

The microdevice was fabricated by machining a 6.0 mm thick, 10 mm × 10 mm polymethyl methacrylate (PMMA) plate (Mitsubishi Rayon, Tokyo, Japan) using computer-aided design and computer-aided manufacturing with a 3D modeling machine (MM-100, Modia Systems, Japan), as shown in Fig. 1b (30, 33). Two wells (2.0 mm diameter and 4.5 mm depth) and a chase between the wells were manufactured on the PMMA plate. However, in the experiment investigating the effect of EGCG addition, the microdevice was prepared with a well diameter twice as large as usual (4.0 mm), so that when the solution was later added, there were no sudden changes in osmotic pressure. Each well had a through-hole in the bottom and Ag/AgCl electrodes set into this hole. A polymeric film made of parylene C (polychloro-*p*-xylylene) with a thickness of 5 μm was patterned with single pores (100 μm diameter) using a conventional photolithography method and then fixed between PMMA films (0.2 mm thick) using an adhesive bond (Super X, Cemedine Co., Ltd., Tokyo, Japan). The films, including the parylene film, were inserted into the chase to separate the wells.

### Preparation of planar lipid bilayer in the microdevice

Planar lipid bilayers were prepared by the droplet contact method (34) using a microdevice. First, PC, DOPC/Chol. (4:1 [w/w]), DOPC/SM/Chol. (5:1:2 [w/w]), or BTLE (lipids/*n*-decane, 10 mg/mL) solution (2.3 µL) was poured into each chamber. Next, PBS buffer solution (4.7 µL) with Aβ42 (final concentration of 10 µM) was poured into both chambers (28, 29). When the planar lipid bilayers ruptured, they were reconstituted as planar lipid bilayers by tracing with a hydrophobic stick between two droplets.

### Channel current measurements and data analysis

The channel current was monitored using a Pico patch clamp amplifier (Tecella, Foothill Ranch, CA, USA) connected to the chambers. Ag/AgCl electrodes were already present in each droplet when the solution was added to the chambers. A constant voltage of +100 mV was applied to the recording chamber, and the ground chamber was grounded. Pore formation in the lipid bilayer allowed ions to pass through the nanopore under the voltage gradient, giving the channel current signals. The signals were detected using an 8-kHz low-pass filter at a sampling frequency of 40 kHz. An analysis of channel current signals and duration time was performed using Clampfit ver. 10.7 (Molecular Devices, San Jose, CA, USA) and Excel (Microsoft, Redmond, WA, USA) softwares. Channel current measurements were conducted at 22 ± 2°C.

### Western blotting

To assess the aggregation states of Aβ42 in the presence of lipid, 9.4-µL samples of Aβ42 (10 µM in PBS) and 4.6-µL lipid (DOPC, DOCP:Chol., DOPC:SM:Chol., or BTLE) were incubated in Protein LoBind Tubes for 2 h at room temperature. Next, 3 µL of a sample buffer solution with reducing reagent (6×) for SDS–PAGE buffer was added to the Aβ42 solution and incubated on ice for 10 min. The Aβ42 was separated in Any kD Mini-PROTEAN TGX Precast Protein Gels (Bio-Rad) at a constant voltage of 150 V for 45 min using a Mini-PROTEAN Tetra Vertical Electrophoresis Cell (Bio-Rad Laboratories, Inc., Hercules, CA, USA). The separated Aβ42 was transferred onto nitrocellulose membranes at 21 A and 25 V for 3 min using the Trans-Blot Turbo Transfer System (Bio-Rad Laboratories). Membranes were blotted for 1 h in a solution of 4% skim milk in PBS-T (phosphate-buffered saline containing 0.05% Tween-20) and washed in PBS-T for 5 min. 82E1 was used as a primary antibody against Aβ42 (1:2,000 in PBS-T, 1 h at room temperature), and HRP-conjugated antimouse IgG was used as the secondary antibody (1:5,000 in PBS-T, 1 h at room temperature). Finally, Aβ42 was detected by chemiluminescence using Immobilon western substrate (Merck Millipore, Tokyo, Japan) with a Las4000 mini (Cytiva, Tokyo, Japan).

### ThT fluorescence measurement

Sixty-microliter samples of Aβ42 (10 µM in PBS) and 30-µL lipid (DOPC, DOPC:Chol., DOPC:SM:Chol., or BTLE) were incubated in Protein LoBind Tubes for 1, 2, 3, 4, 5, 7, and 8 h at room temperature. The aggregation of Aβ42 was monitored by the ThT assay using a Varioskan Flash reader (Thermo Fisher Scientific, Inc., Waltham, MA, USA). The powdered ThT was dissolved in 0.1 N HCl to a weight ratio of 0.5% and diluted with PBS buffer to a final concentration of 20 µM. Fifty microliters of ThT were added to 50-µL samples of Aβ42. Fluorescence at 486 nm was measured at an excitation wavelength of 450 nm. The blank fluorescence (PBS buffer) was subtracted.

### AFM imaging for Aβ42 aggregation

For imaging the aggregation of Aβ42, 20-µL samples of Aβ42 (10 µM in PBS) and 10-µL lipid (DOPC, DOPC/Chol., DOPC/SM/Chol., or BTLE) were incubated in Protein LoBind Tubes for 2 h at room temperature. Two-microliter samples of Aβ42 solution were deposited onto a freshly cleaved mica surface. After incubation for 10 min at room temperature, the mica surface was gently washed with PBS buffer. The AFM imaging of Aβ42 aggregations was performed in PBS buffer using a high-speed AFM system (BIXAM, Olympus, Tokyo, Japan) with a silicon nitride cantilever (resonant frequency = 0.8 MHz, spring constant = 0.1 N/m, EBD tip radius < 10 nm; USC-F0.8-k0.1-T12; Nanoworld, Neuchâtel, Switzerland) (35). The images of 320 × 240 pixels were obtained at a scan rate of 0.2 fps for static images.

### Addition of EGCG to Aβ42 embedded in the lipid bilayer

The powdered EGCG was dissolved in DMSO to a final concentration of 10 mM and stored at −80°C. Immediately before use, samples were diluted in PBS buffer solution to 100 µM.

For measurements on EGCG addition, the microdevice with larger wells was used, with 4 µL of lipid solution poured into each chamber and 16 µL of Aβ42 (12.5 µM) solution subsequently added to form a lipid bilayer. Approximately 30 min after the appearance of the Aβ42 signal, 4 µL of EGCG (500 µM; control: 4 µL PBS buffer) was added, and the measurement was continued for another 30 min. The final concentrations of Aβ42 and EGCG were 10 and 100 µM, respectively.

## Results

### Comparison of current signals between the Aβ42 monomer and oligomer

**Fig. 2.**
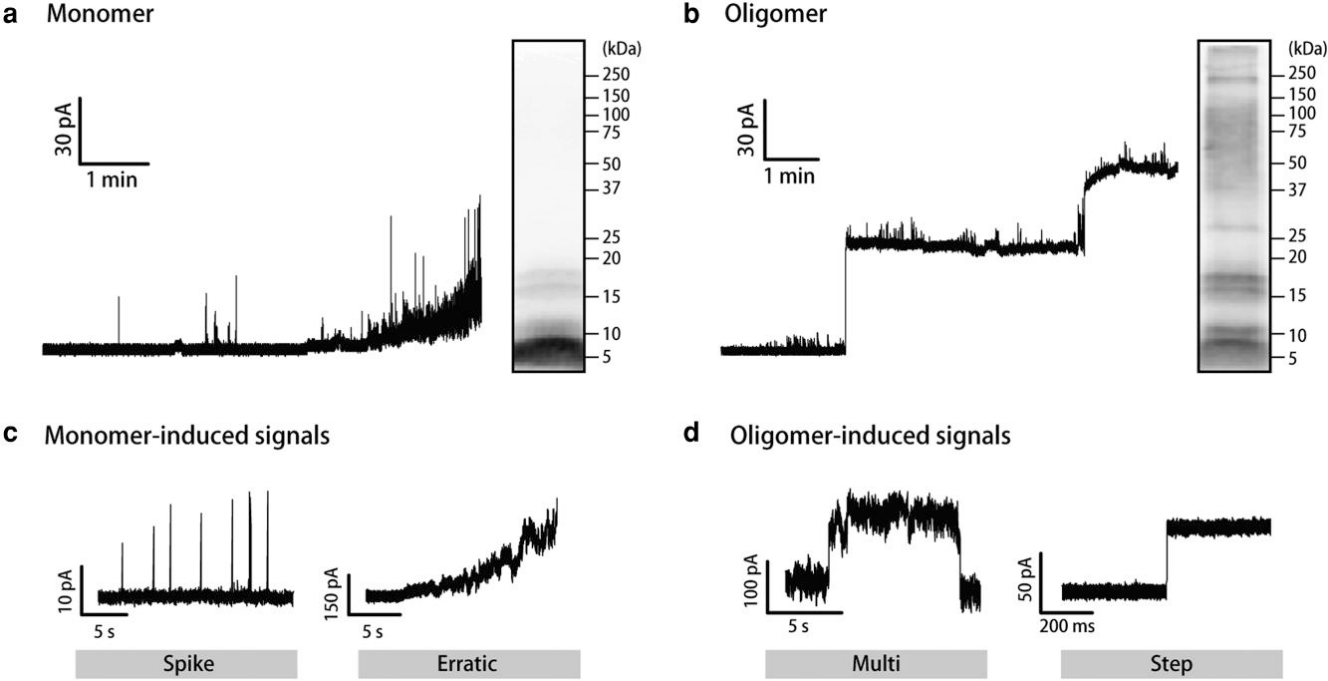
a) Typical signal for the measurement of the Aβ42 monomer (left) and WB analysis of the Aβ42 monomer (right). b) Typical signal for the measurement of the Aβ42 oligomer (left) and WB analysis of the Aβ42 oligomer (right). Both current measurements were performed using 10 µM of Aβ42, a PC membrane, and an applied voltage of 100 mV. The typical signal shapes of the c) spike and erratic signals and d) multilevel and step signals. The spike and erratic signals were assigned to monomer-induced signals, and the multilevel and step signals were assigned to oligomer-induced signals.

We initially elucidated the difference between the channel current signals for the Aβ42 monomer and oligomer. The Aβ42 monomer was prepared, and the monomeric state was confirmed using WB. A deep band at around the 4–8 kDa region was observed, indicating the formation of the monomer (*M_w_* = 4.5 kDa) configuration, and the peak dimer band was also observed at around the 10 kDa region as shown in Fig. 2a. The channel current signals in the DOPC membrane represented in Fig. 2a increase gradually with a noise-like current shape. On the other hand, the oligomer, which was prepared from the monomer state (see Experimental section), presented smeared and ladder-shaped bands in the high-molecular-weight region in the WB (Fig. 2b). The molecular weight of the Aβ42 oligomer, which has strong cytotoxicity, is reported as 17 kDa (4-mer) to 90 kDa (20-mer). Our prepared oligomer showed similar molecular weight in the WB, and step-like currents were observed in the case of oligomer current measurements (Fig. 2b).

We have previously proposed the classification of the current signals of AMPs, which form the α-helical structure, into mainly four types: spike, erratic, multilevel, and step (Fig. 2c and d) (29). These four types of current signals were assigned to the membrane penetration or pore-formation models that have already been proposed based on spectroscopic experiments. We also assigned four current signals from the Aβ42 monomer/oligomer to four models: direct penetration model (spike signal), random disruption model (erratic signal), toroidal model (multilevel signal), and barrel-stave model (step signal) as depicted in Fig. 2c and d. Because the Aβ42 and other β-sheet peptides are known to form the toroidal or barrel-stave model pore in the lipid membrane (36), we consider that the observed step current reflects the rigid pore formation of Aβ42 oligomers. The average duration of the current step was 9.8 ± 2.7 min, encompassing the time required for Aβ42 oligomers to reach the membrane and the subsequent pore formation (25). In contrast, we have not observed the current signals of Aβ40 in our conditions.

Summarizing these results, the monomer mostly shows the spike and erratic signals, and the oligomer mainly shows the multiple and step signals, reflecting the rigid channel formation in the lipid membrane. Therefore, in this study, we defined the spike and erratic signals as “monomer-induced signals”; the other two signals were assigned to “oligomer-induced signals.”

### Real-time observation of Aβ42 oligomerization in the lipid membrane

**Fig. 3.**
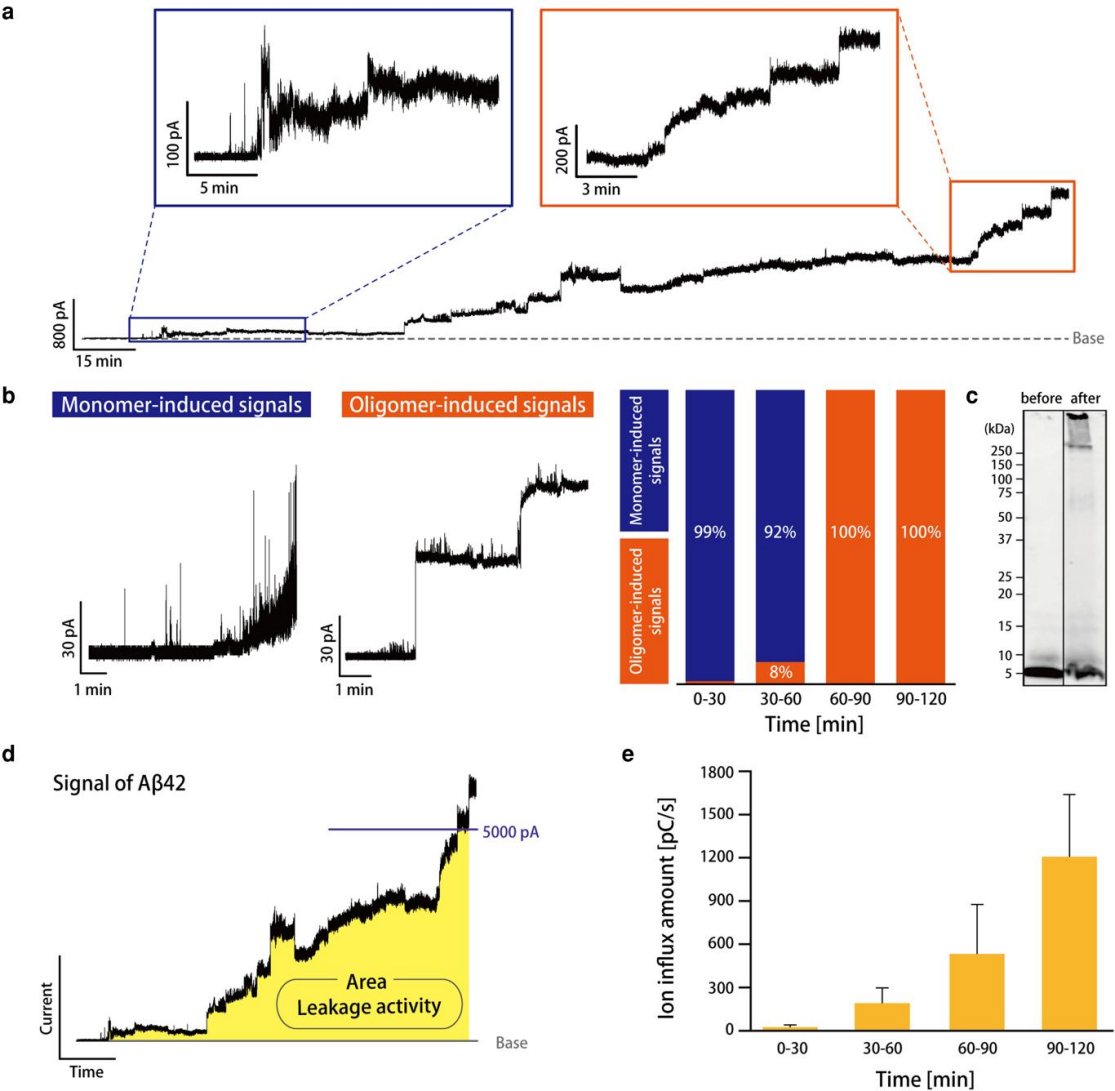
a) Signal obtained from the channel current measurements of Aβ42 (10 µM) in a PC membrane for 2 h at room temperature (applied voltage: 100 mV). b) The ratio of monomer-induced and oligomer-induced signals is shown every 30 min. In each time zone, the number of oligomer-induced and monomer-induced signals was counted, and the ratio of the number of each signal was calculated (*n* = 5; *n*, the number of 2-h measurements). c) The WB analysis of Aβ42 before measurement (before) and Aβ42 collected after 2 h of measurement in the droplet chamber (after). The measurement conditions were as follows: 10 µM of Aβ42 in a PC membrane and an applied voltage of 100 mV. d) Definition of ion flux: ion flux was calculated from the area between the baseline and the current amplitude as colored in yellow. e) Value of ion flux every 30 min (*n* = 5; *n*, the number of 2-h measurements).

We next attempted to observe the oligomerization process of the Aβ42 monomer in the DOPC membrane by the current signal transition. To determine the time of measurement for the oligomerization, we performed fluorescence measurements using ThT, which fluoresces when binding to the Aβ42 fibrils. The fluorescence intensity of ThT was measured in samples incubated at 37°C for 1, 2, 3, 4, 5, 7, and 8 h, respectively, and the intensity became higher in samples incubated for >4 h (Fig. S1). These results imply that Aβ42 forms mature fibrils via the oligomerization state after 4 h in these conditions. Next, we investigated the transition of the current signal from the monomer-induced signal to the oligomer-induced signal, with a typical current recording shown in Fig. 3a. The monomer-induced signals were observed at the early stage (0 to 60 min), and thereafter the oligomer-induced signals, such as step signals, appeared in the current trace (Fig. 3b). After the measurements, we took the recording solution and checked the oligomerization of the Aβ42 monomer in the recording chamber by WB, which resulted in gels with bands in the high-molecular-weight region (over 250 kDa, calculated to be >55 mer, at stacking gel; Fig. 3c). The aqueous solution in the recording chamber is surrounding the lipid monolayer, and it has been reported that such a lipid environment may induce the oligomerization of the Aβ42 monomer. We also checked the oligomerization of the Aβ42 monomer in a protein low-bind tube that has aqueous and lipid/oil phases, similar to the recording condition. After incubating for 2 h, several bands appeared between 75 and 250 kDa, giving a similar result to the samples extracted from the recording chamber (Fig. S2).

The entire current signals were classified into the “monomer-induced” or “oligomer-induced” signal in accordance with previous classification methods (28, 29), and the appearance ratio of each signal was calculated every 30 min, as shown in Fig. 3b. The monomer-induced signal was observed mainly in the initial hour; after that, most of the signals suddenly transitioned to the oligomer-induced signal. This rapid transition occurred at around 50 min (Fig. S3). This time was not contradictory to the report by Choucair et al. (37) that the small aggregates of Aβ42 started to accumulate on the domains approximately after 85–90 min of incubation with DOPC/1,2-dipalmitoyl-*sn*-glycero-3-phosphocholine (DPPC) bilayers. Next, the ion flux of Aβ42 channels/defects was estimated. This value reflects the peak activity of ions through the transmembrane channel. The ion flux is the number of ions flowing into the channel per unit time, and it is calculated from the area of the signal up to the limiting current value (5,000 pA; Fig. 3d) (30). Figure 3e shows the results of the ion flux every 30 min. The value increased with time, suggesting that many or large channels/defects were formed.

### The channel-forming and aggregation activities of Aβ42 in different lipid compositions

We next investigated the channel formation and aggregation activities of Aβ42 (10 µM) in the four different compositions of lipid membrane: [PC], [PC:Chol.], [PC:SM:Chol.], and [BTLE (porcine brain extraction)]. The [PC] and [PC:Chol.] (=4:1 w/w) membranes imitate a mammalian cell membrane. The [PC:SM:Chol.] (=5:2:1 w/w) and [BTLE] membranes mimic the nerve cell membrane, and the [PC:SM:Chol.] membrane should form lipid raft (domain) structures in artificial lipid membranes (38). The effect of the lipid raft can be examined using this system because Aβ42 is prone to aggregate around a lipid raft structure (39). We estimated the channel-forming and aggregation activities using the channel current recordings, WB, AFM, and the fluorescent intensity of ThT.

**Fig. 4.**
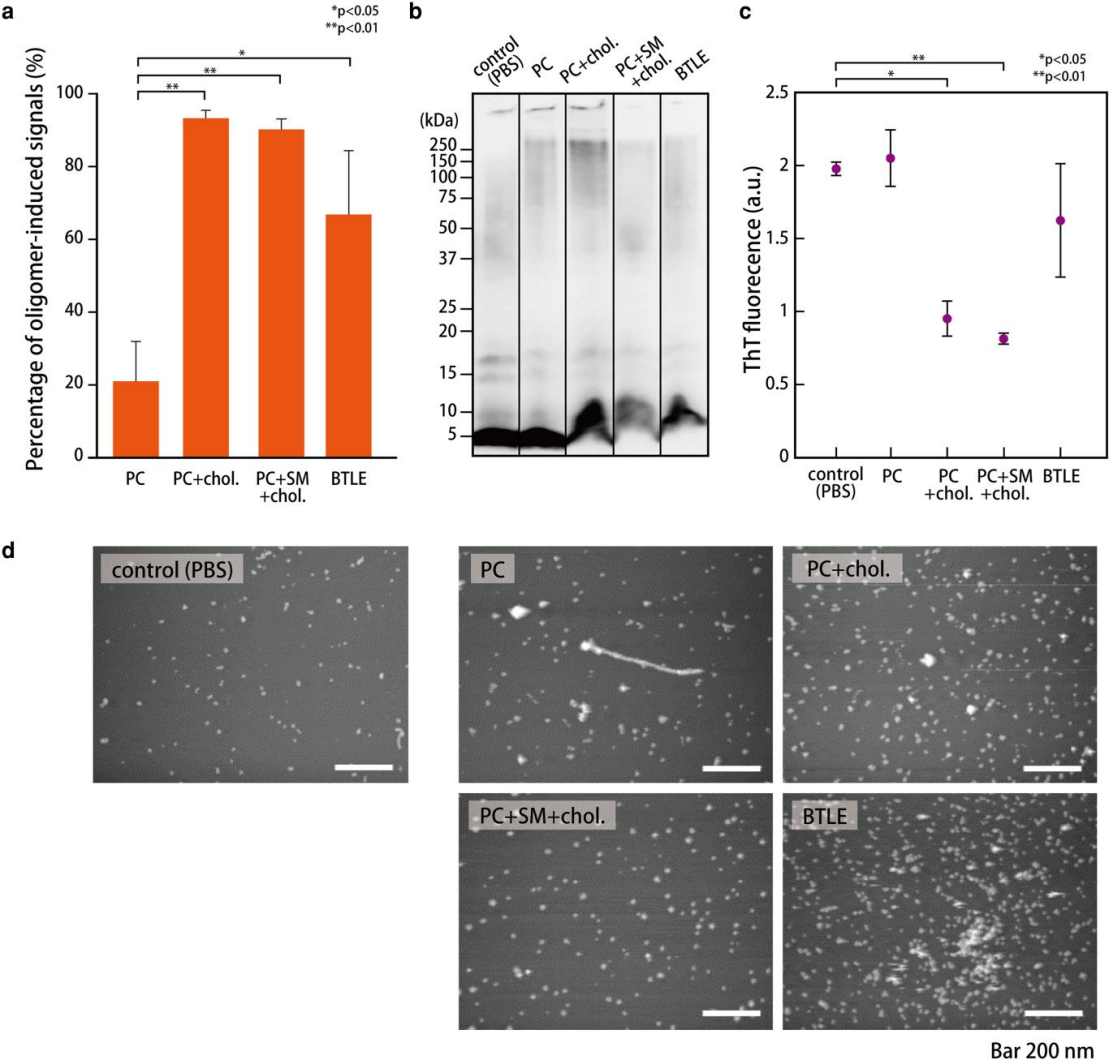
For each lipid composition, a) the ratio of oligomer-induced signals. Each signal obtained from the 2-h measurement was classified into a monomer-induced or oligomer-induced signal. The average value of the percentage of oligomer signals analyzed for each signal was calculated (*n* = 5–6; *n*, the number of 2-h measurements). Statistical analysis was performed using the Dunnett test. b) The WB analysis of Aβ42. c) Fluorescence intensity of ThT bound to Aβ42 fibrils (*n* = 3). Statistical analysis was performed using the Holm test. d) The AFM image of Aβ42. The white areas are Aβ42. Scale bar: 200 nm. The concentration of Aβ42 was unified to 10 µM in all experiments.

For the channel current recordings, the appearance ratio of the oligomer-induced signals from all observed signals over 2 h for the four different membrane compositions is shown in Fig. 4a. The ratio follows the order: [PC:Chol.] ≃ [PC:SM:Chol.] > [BTLE] >> [PC]. To confirm the aggregation state of Aβ42 in each membrane composition, the Aβ42 monomer was incubated for 2 h in a protein low-bind tube with the aqueous/lipid oil solution of each membrane composition. The oligomerization/aggregation states were checked by WB, AFM, and fluorescent measurement using ThT. In the WB for each lipid membrane composition, smeared bands were mainly observed ranging from 75 to 250 kDa, which is an oligomer region (Fig. 4b). Among them, the stronger bands in this oligomer region were observed in the [PC:Chol.] composition when using a semiquantitative analysis of the images (using ImageJ software as shown in Fig. S4). In the ThT measurements, the order of the intensity was [PC] ≃ [BTLE] > [PC:Chol.] ≃ [PC:SM:Chol.] (Fig. 4c). The lower intensity in [PC:Chol.] and [PC:SM:Chol.] conditions may suggest reduced protofibril formation. In the AFM observations, nanosized aggregated particles were observed for each condition (Fig. 4d), and the number of particles and their area were analyzed in each image (using ImageJ as shown in Fig. S5). The average area of spherical Aβ42 aggregates was not contradictory with a previous study with DOPC/DPPC bilayers (see more detail in Fig. S5) (37). Although a few fibril-like structures were observed in the [PC] composition, Aβ42 incubated with the other compositions showed only spherical aggregates. The number of Aβ42 aggregates in 1 µm² was the largest for the [PC:Chol.] composition. In the [BTLE] composition, the number of aggregates and their area were larger than that for the other compositions.

### Analysis of the channel size and ion flux of Aβ42 oligomers

**Fig. 5.**
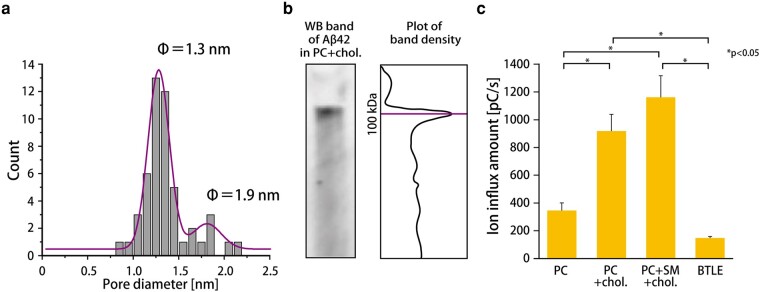
a) Histogram of diameter values of the Aβ42 channel in the [PC:Chol.] membrane calculated from the Hille equation. Histograms under all lipid conditions are shown in Fig. S6. b) The WB analysis. The band of Aβ42 (10 µM) in [PC:Chol.] (left) and the plot of band density using *ImageJ* (right). The photo of the whole band is shown in Fig. S7. c) Ion flux value of Aβ42 (10 µM) for each lipid composition; the average of the ion flux values was calculated from the signals obtained over a 2-h measurement (applied voltage: 100 mV). The ion flux of each signal was the average of the ion flux values every 5 min (*n* = 5–6; *n*, the number of 2-h measurements). Statistical analysis was performed using the Steel–Dwass test.

We next estimated the size of channels formed by the Aβ42 oligomers using the channel conductance of the step signals. The diameter of the channel was calculated using the Hille equation:


$$R=\frac{V}{I}=\left(L+\frac{\pi d}{4}\right)\frac{4\rho }{\pi d^{2}}$$

where *R* is resistance, *V* is applied voltage (V), *I* is current (A), *L* is the length of the channel (m), *ρ* is ion conductivity (Ω m), and *d* is the diameter of the channel (m). Figure 5a shows the histogram of the channel diameter of Aβ42 for the [PC:Chol.] composition: 1.3 and 1.9 nm are the main peak and subpeak, respectively. The channel size in the other membrane compositions is listed in Table 1. In the case of the [PC] composition, the channel diameter could not be calculated because the step signal was not observed. Next, we estimate the number of assembling monomers of Aβ42, combined with the data from WB. The observed bands in the [PC:Chol.] composition were analyzed by their color intensity, resulting in a single large peak at around 100 kDa (Fig. 5b). The number of the assembling monomers can be calculated by the molecular weight of the monomer (4.5 kDa); this gives a 22-mer.

**Table 1.** The estimated diameter of the Aβ42 channel under membrane conditions for each lipid composition.

Membrane	Diameter main peak (40)	Diameter subpeak (40)
[PC:Chol.]	1.3	1.9
[PC:SM:Chol.]	1.2	3.7, 6.6
[BTLE]	1.3	Not determined

The diameter was calculated from the Hille equation.

The ion flux in each membrane composition is shown in Fig. 5c. The order of the ion flux is [PC:SM:Chol.] > [PC:Chol.] > [PC] > [BTLE].

### Inhibition of the oligomerization of Aβ42 in lipid membranes using EGCG

**Fig. 6.**
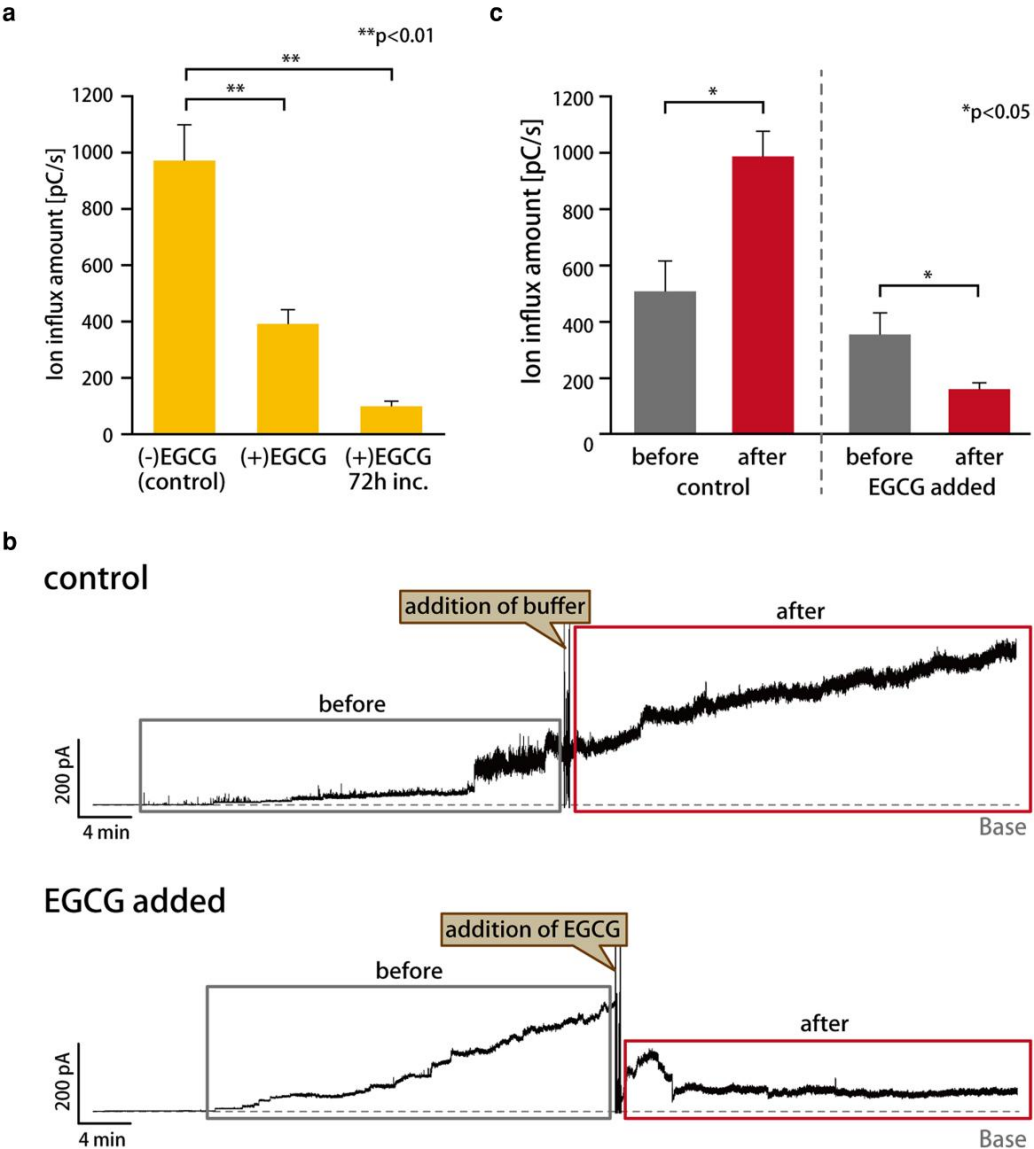
a) Ion flux value of Aβ42 (10 µM) for each condition; the average of the ion flux values was calculated from the signals obtained over 2-h measurements. The ion flux of each signal was the average of the ion flux values every 5 min (*n* = 4–5; *n*, the number of 2-h measurements). Statistical analysis was performed using the Steel–Dwass test. b) The signals were observed when the solution (PBS buffer or 100 µM of EGCG solution) was added after the appearance of the Aβ42 signal. “before” was the signal before the solution was added, and “after” was the signal after the solution was added. Less than 5% of the DMSO solution (final conc.) did not interfere with the lipid membrane in the current measurements (Fig. S9). c) Comparison of ion flux values of Aβ42 before and after the addition of solution with Welch's t test. The ion flux value of the signal was calculated for 25 to 40 min before and after adding the solution, respectively. The ion flux of each signal was the average of the ion flux values every 2 min (*n* = 3–4; *n*, the number of measurements).

We next attempted to estimate the inhibition effect of the Aβ42 oligomerization in our lipid bilayer system. EGCG, which is the main catechin in green tea, was used in this experiment because the high activity of the aggregation inhibition of Aβ42 has previously been reported (36, 41). Based on the previous experimental conditions, we conducted and measured the current signal of the Aβ42 monomer with 100 µM EGCG, incubating for 2 and 72 h in the [PC:Chol.] membrane composition. The ion flux after incubation with EGCG decreased dramatically, as depicted in Fig. 6a. Particularly, for the 72 h incubation, the ion flux was reduced by around 10% compared with the control experiment. EGCG itself did not show any current signal (Fig. S8). To confirm that this phenomenon is also observed when Aβ42 is embedded in the lipid membrane, we added EGCG after observing (around 30 min) the oligomer-induced current signals. The current signal also decreased after adding EGCG, as shown in Fig. 6b. In addition, the ion flux was also reduced upon EGCG addition vs. the control experiment (Figs. 6c and S8).

## Discussions

### Real-time and long-term observation of Aβ42 oligomerization using the lipid bilayer system which provides a useful tool for observing Aβ42 dynamics with lipid membranes

Oligomerization, or crystallization prenucleation, is notoriously difficult to detect experimentally. They are expected to have very short lifetimes, and the populations are inherently limited. To date, although ThT and AFM experiments have used alternate approaches to monitor the kinetics or dynamics of oligomerization, it has remained a challenge (42, 43). Several studies have reported the channel current measurement of Aβ42 using a planar lipid bilayer, which is formed with the Montal–Mueller or painting methods (15, 25). Although these methods are well known as the conventional methods for lipid bilayer formation, the bilayer mechanical stability has been a serious issue for long-term experiments. The previous reports therefore showed mainly the channel conductance and did not mention the kinetic behavior of Aβ42, such as the monomer-to-oligomer transition. This is probably due to the issue of the low mechanical stability of the lipid membrane. On the other hand, in this study, we conducted all the channel current measurements for 2 h and compared them with each dataset, including the ion flux and signal classification. The ratio of the oligomer-induced signals and the ion flux values increased with time (Fig. 3b and d), suggesting that Aβ42 formed channels upon oligomerization in the membrane. This result is also supported by the WB; the bands appeared in the oligomer region after the 2-h measurement (Fig. 3c). These results suggest that we observed the process of aggregation of the Aβ42 monomer into the oligomer and its channel formation around 60 min. Moreover, the lipid molecules that surround the aqueous phase should also have a strong effect on the oligomerization, as previously mentioned.

### Chol. induces Aβ42 oligomerization in the lipid membrane

Using our 2-h measurements, we can estimate the real-time Aβ42 oligomerization with a lipid membrane. We next investigated what the most significant molecules were for Aβ42 oligomerization by using different membrane compositions: [PC], [PC:Chol.], [PC:SM:Chol.], and [BTLE (porcine brain extraction)]. Through several experiments such as current recording, WB, ThT, and AFM observation, we conclude that Chol. is the most significant factor for the Aβ42 oligomerization in the membrane. The ratio of the oligomer-induced signal in both [PC:Chol.] and [PC:SM:Chol.] conditions are high (Fig. 4a), as in previous studies, which reported that Chol. may induce the frequency of channel formation (44–46). It has previously been reported that the cytotoxicity induced by Aβ42 drastically increased in the presence of Chol. in lipid membranes (47, 48). Similarly, the ion flux, which reflects the pore-forming activity, was increased in the presence of Chol. The other experiments also support the strong effect of Chol.; in the [PC:Chol.] condition, bands are observed ranging from 75 to 250 kDa in the WB (Figs. 4b and S4), and there are many aggregation particles under AFM observation (Figs. 4d and S5) (24, 49). Besides, the intensity of ThT fluorescence in the [PC:Chol.] condition is lower than that for the other conditions (Fig. 4c), suggesting reduced protofibril formation (50).

The kinetic model of the oligomerization and fibrillization of Aβ42 has recently been proposed as [monomer] ⇆ [oligomer] ⇆ [fibril] (51). Also, it has already been reported that the Aβ42 has a Chol.-binding domain (Aβ22–35) (52). Combining these facts, the Aβ42 monomer may bind to Chol. and form the “oligomer channel”; however, it may be difficult for the formed oligomers to proceed to form the fibril structure when the lipid membrane has Chol., as summarized below:

[PC:Chol.] or [PC:SM:Chol.]: [monomer] → [channel oligomer]

[PC] or [BTLE]: [monomer] → (protofibril) → [fibril]

### The channel size and ion flux of the Aβ42 oligomer

The Aβ42 monomers assemble to form the oligomer channel in a planar lipid bilayer, resulting in the appropriate current signals (53). The size of the channel can be estimated by the conductance of the step signals for each membrane condition (Fig. 5). In the case of the PC + Chol. membrane, the diameter is 1.3 nm, with monomers assembling as the 22-mer. Although the histogram of the channel diameter showed several different peaks depending on the lipid composition (Table 1), the highest peak was commonly observed around 1.3 nm. This channel size may be the most stable state to assemble the Aβ42 monomers in the lipid membrane. Besides, this value corresponds well with previous reports estimated by patch clamping of living nerve cells (1.7, 2.1, and 2.4 nm) (25) and MD simulation (1.9 nm, 18-mer) (27).

The ion flux may directly reflect the membrane disruption–induced cytotoxicity. The value of the ion flux in the [PC:Chol.] and [PC:SM:Chol.] membranes was relatively high (Fig. 6c). Also, the amount of the oligomer-induced current signals increased in these systems. Combining these results, it seems that the channel-forming activity of Aβ42 is high in the [PC:Chol.] and [PC:SM:Chol.] membranes. On the other hand, the ion flux in the [BTLE] membrane was low, while the binding affinity between Aβ42 and the membrane may be high due to [BTLE] consisting of a negatively charged PS. While the reason for the low ion flux in the [BTLE] membrane is still under consideration, Aβ42 formed the channel with only a 1.3-nm diameter; therefore, the amount of ion flux was relatively low.

### Inhibition of the oligomerization when adding EGCG

Since the ion flux decreased with the prolonged incubation of Aβ42 with EGCG (Fig. 6a), EGCG may inhibit the channel formation of the Aβ42 oligomers. DMSO, used as a solvent for EGCG, was also present in the measuring solution prior to the addition of EGCG, suggesting that DMSO is less likely to inhibit the channel formation of Aβ42. Furthermore, the flux also decreased even when the EGCG was added after inserting the Aβ42 monomers into the lipid membrane (Fig. 6b and c). These results suggest that EGCG inhibits the oligomerization of Aβ42 not only in the aqueous phase but also in the membrane. We therefore considered the configuration of Aβ42 in the lipid bilayer (54, 55).

The EGCG molecule binds to H14, Y10, and K28 of a tetramer of Aβ42 with π–π (H14, Y10) or cation–π (K28) interaction in solution, resulting in the inhibition of aggregation (31, 56). On the other hand, in the case of inhibition in the membrane phase, EGCG may bind the outer-membrane region of Aβ42 from the aqueous phase, because EGCG is relatively insoluble in the hydrophobic lipid phase. The MD simulation of Aβ42 in the lipid membrane depicts that Aβ42 monomers assemble to form β barreled structures, and the hydrophobic N termini are exposed to the outer-membrane region (57). Also, the H14 and Y10 are set near the N terminus. Therefore, EGCG may bind around the N terminus to collapse the oligomerization state.

## Conclusion

In summary, we prepared lipid bilayers that imitate neuronal cell membranes and evaluated the channel-forming activity of Aβ42 in the membranes by electrophysiological methods. Current measurements demonstrate that the Aβ42 aggregated from monomers to oligomers and formed channels in the membrane. In addition to the current measurements, the results of WB analysis, ThT fluorescence intensity, and AFM observation suggest that Chol. was a key component that promoted the Aβ42 oligomerization, resulting in the formation of the channel structure. The current measurements and WB bands showed that the Aβ42 oligomers formed channels with a diameter of ∼1.3 nm (22 mer), consistent with previous studies (27). This size of oligomer forms a relatively stable channel in the lipid membrane and would induce the cytotoxicity. Furthermore, current measurements using the inhibitor EGCG showed that the addition of EGCG from outside of the membrane decreased the channel-forming activity of both the Aβ42 monomer and oligomer.

These results shed light on the monomer-to-oligomer transition and the channel formation of Aβ42 with several different types of lipid membranes, such as a composition imitating the nerve cell membrane. Although the relationship between AD and Aβ42 oligomerization is still unclear and involves more complex factors, the current measurement used in this study is a promising method for understanding the molecular assembly mechanism while imitating several types of lipid systems.

## Supplementary Material

pgad437_Supplementary_Data

## Data Availability

All data are included in the manuscript and/or Supplementary material.
