# Supplementary material for: Real-time monitoring of the amyloid β1–42 monomer-to-oligomer channel transition using a lipid bilayer system
Source: PNAS Nexus. 2023 Dec 14;3(1):pgad437. doi: 10.1093/pnasnexus/pgad437 (PMC10753159; doi:10.1093/pnasnexus/pgad437)
Supplement: pgad437_Supplementary_Data [file pgad437_supplementary_data.pdf]

Supporting Information

Real-time monitoring of the Aβ42 monomer-to-oligomer channel transition using a lipid bilayer system

Yuri Numaguchi[1,†], Kaori Tsukakoshi[1†], Nanami Takeuchi[1], Yuki Suzuki[2], Kazunori Ikebukuro[1], and Ryuji Kawano[1]

[1]*Department of Biotechnology and Life Science, Tokyo University of Agriculture and Technology, Tokyo, JAPAN*

[2] *Department of Chemistry for Materials, Graduate School of Engineering, Mie University, Mie, Japan*

[†] These authors contributed equally to this work.

*Corresponding author (rjkawano@cc.tuat.ac.jp)

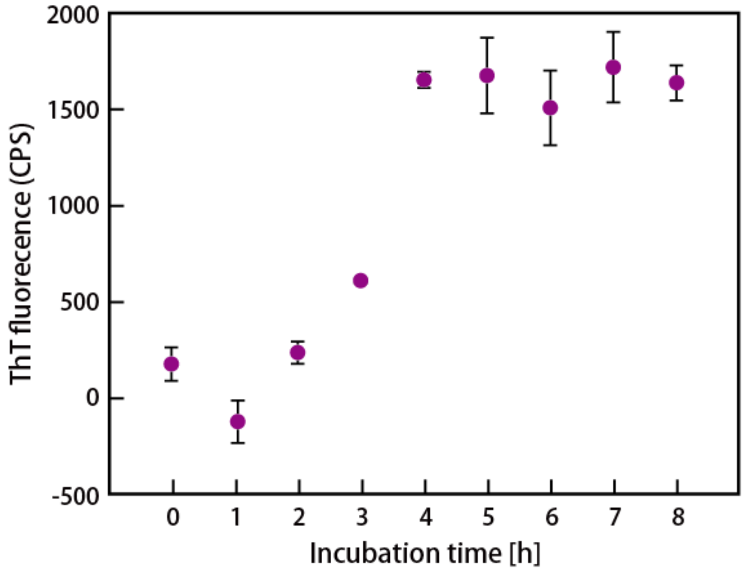

Fig. S1 Fluorescence intensity of ThT bound to Aβ42 fibrils incubated at 37°C for each timepoint (n=3).

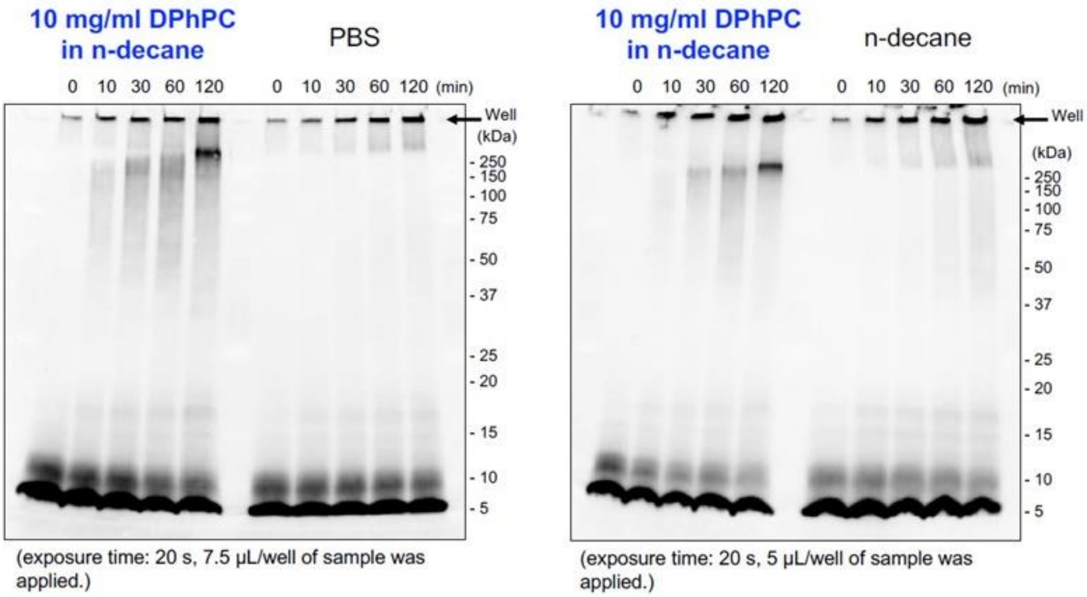

Fig. S2 WB analysis of Aβ42 incubated with or without lipid/*n*-decane solution. Aβ42 (10 μM) with or without PC solution was incubated in Protein LoBind tubes for 2 hours at room temperature.

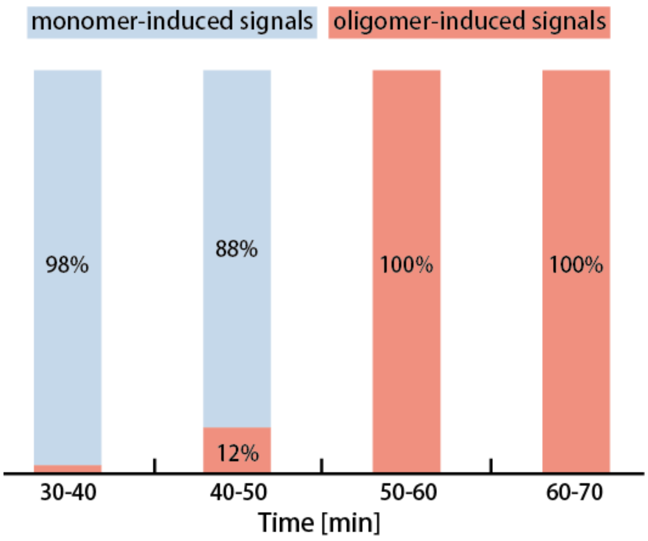

Fig. S3 Ratio of oligomer-induced and monomer-induced signals every 10 minutes within 30-70 minutes after the start of measurement. For each time slot, the number of oligomer-induced and monomer-induced signals were counted and the ratio for each signal was calculated (n=5, n: the number of data within 2 hours' measurement). The measurement conditions were: 10 μM of Aβ42 in a PC membrane and an applied voltage of 100 mV.

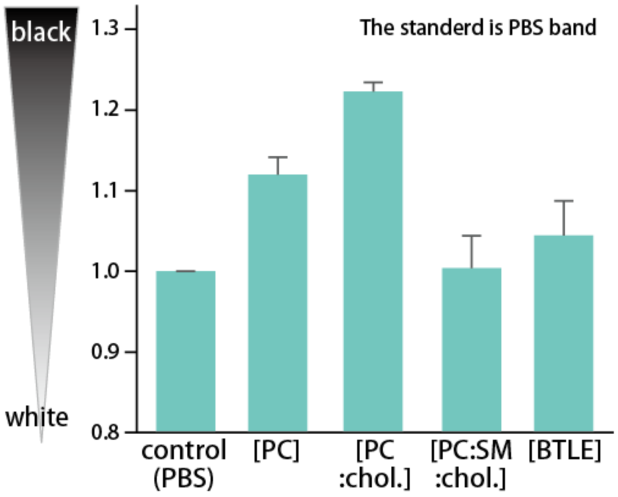

Fig. S4 The band density of WB in the molecular weight range of 75-250 kDa. Using *imageJ*, the gray value of the band with a molecular weight in the range of 75 to 250 kDa was analyzed and the reciprocal of the value calculated. The reciprocal of the gray value of PBS was defined as the standard of 1, whilst the values for each lipid composition were compared (n=3).

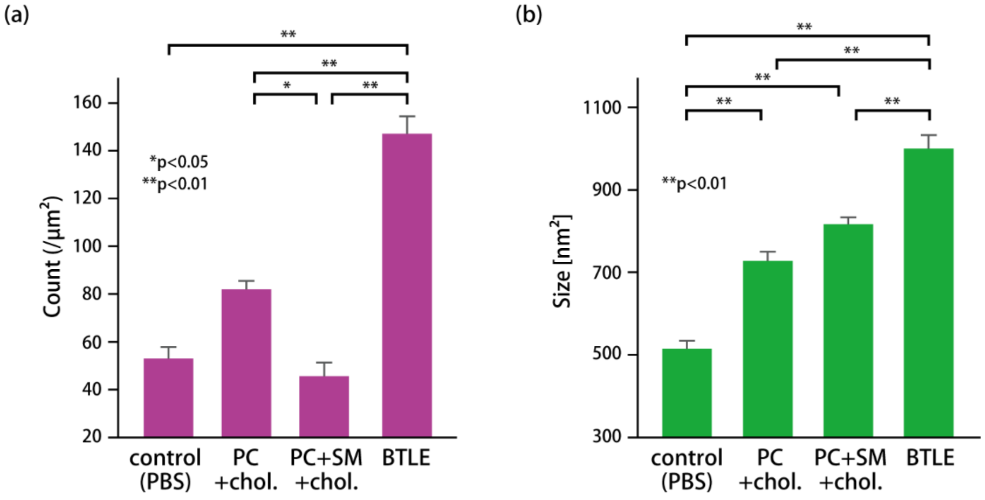

Fig. S5 (a) Number of Aβ42 aggregates. Spherical agglomerates were counted and the number of agglomerates present per µm² was calculated (n=4-5). (b) The average area of spherical Aβ42 aggregates (n=4-5). Statistical analysis for both data was performed using Tukey-Kramer test. Relatively smaller values in our results comparing with a previous study with DOPC/DPPC bilayers(1) are probably due to the use of a cantilever with a sharp, electron beam deposited (EBD) tip, which minimize tip convolution effects in AFM observation.

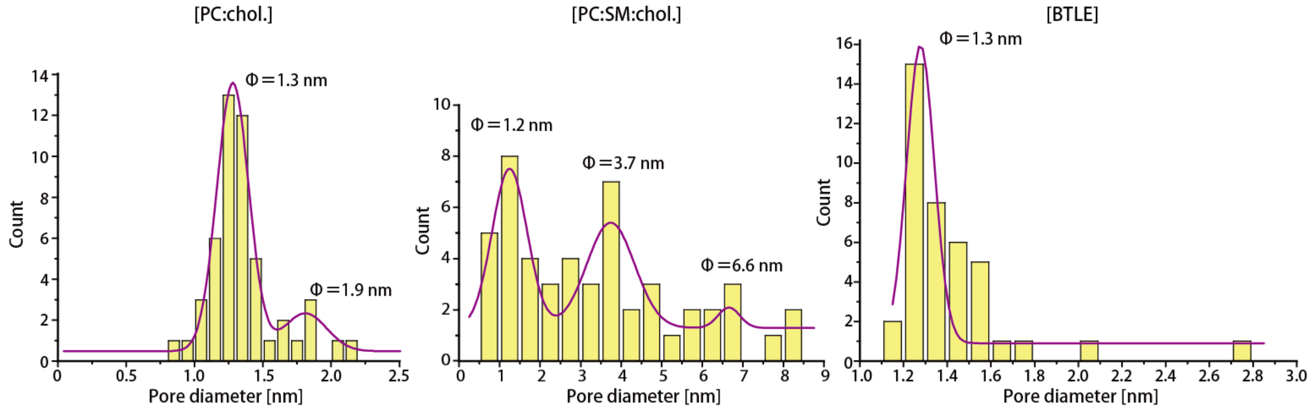

Fig. S6 Histogram of diameter values of the Aβ42 channel in [PC:chol.], [PC:SM:chol.], and [BTLE] membrane calculated using the Hille equation.

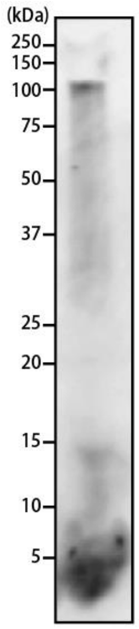

Fig. S7 WB analysis of Aβ42 (10 μM) incubated with [PC:chol.] for 2 hours at room temperature.

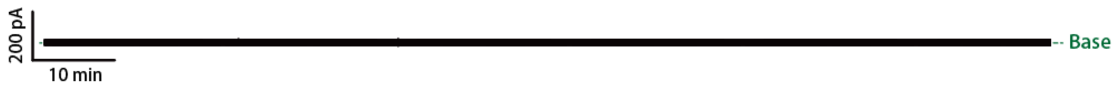

Fig. S8 Signal of EGCG (100 µM) only in [PC:chol.] membrane. No signal was observed for 2 hours (n=3).

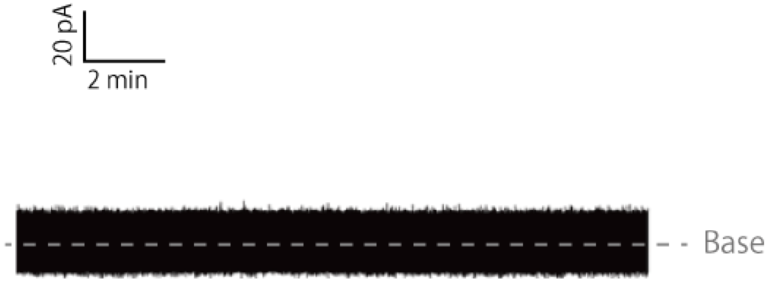

Fig. S9 Signal of 5% DMSO with a PC membrane at +100 mV.

Reference

1. A. Choucair, M. Chakrapani, B. Chakravarthy, J. Katsaras, L. J. Johnston, Preferential accumulation of Aβ(1-42) on gel phase domains of lipid bilayers:: An AFM and fluorescence study. *Biochimica Et Biophysica Acta-Biomembranes* **1768**, 146-154 (2007).
